# HLA-DQB1*03:01 and HLA-DQA1*05:05 as key genetic determinants of infliximab response and immunogenicity in Japanese patients with inflammatory bowel disease

**DOI:** 10.1007/s00535-026-02354-z

**Published:** 2026-02-06

**Authors:** Ryuya Osaka, Takeo Naito, Seik-Soon Khor, Yoichi Kakuta, Yosuke Kawai, Masao Nagasaki, Hiroshi Meguro, Hideya Iwaki, Daisuke Okamoto, Hiroshi Nagai, Yusuke Shimoyama, Rintaro Moroi, Hisashi Shiga, Yoshitaka Kinouchi, Atsushi Masamune

**Affiliations:** 1 Division of Gastroenterology, Tohoku University Graduate School of Medicine, 1-1 Seiryo, Aoba, Sendai, 980-8574 Japan; 2 Genome Medical Science Project, National Center for Global Health and Medicine, Tokyo, Japan; 3 Singapore Centre for Environmental Life Sciences Engineering, Nanyang Technological University, Singapore, Singapore; 4 Division of Biomedical Information Analysis, Medical Research Center for High Depth Omics, Medical Institute of Bioregulation, Kyushu University, Fukuoka, Japan; 5 Center for Genomic Medicine, Graduate School of Medicine, Kyoto University, Kyoto, Japan; 6 Student Health Care Center, Institute for Excellence in Higher Education, Tohoku University, Sendai, Japan

**Keywords:** Infliximab persistence, Anti-drug antibody, Personalized therapy, HLA-DQB1*03:01, Japanese

## Abstract

**Background:**

Specific human leukocyte antigen (HLA) genotypes, particularly HLA-DQA1*05, have been proposed as predictors for infliximab (IFX) treatment response and immunogenicity in Western populations. However, the evidence regarding the effect of HLA-DQA1*05 remains limited in East Asian populations, including in Japan. Moreover, HLA-DQA1*05 frequency differs substantially from those in Western populations. Comprehensive analyses of the association between HLA alleles and IFX treatment outcomes may contribute to the identification of novel prognostic markers for IFX therapies.

**Methods:**

We retrospectively analyzed 301 biologic-naïve Japanese patients with inflammatory bowel disease (IBD). IFX persistence was assessed at both 2-digit and 4-digit HLA allele resolutions, and associations with anti-drug antibody levels at 1 year after the initiation of IFX therapy were evaluated.

**Results:**

At the 2-digit resolution analysis, HLA-DQB1*03 (hazard ratio [HR] = 2.39, *p* = 1.89E-06) and HLA-DQA1*05 (HR = 1.99, *p* = 3.91E-04) were significantly associated with early IFX discontinuation. At the 4-digit resolution analysis, HLA-DQB1*03:01 (HR = 2.03, *p* = 9.42E-05) and HLA-DQA1*05:05 (HR = 2.18, *p* = 4.42E-05) showed similar associations. All HLA-DQA1*05:05 alleles formed haplotypes with HLA-DQB1*03:01. Importantly, HLA-DQB1*03:01 was also associated with early discontinuation of IFX even when it formed haplotypes with alleles other than HLA-DQA1*05:05. Both HLA-DQB1*03:01 and HLA-DQA1*05:05 were significantly associated with elevated anti-drug antibody levels (*p* = 3.23E-03 and 3.54E-03, respectively).

**Conclusions:**

HLA-DQB1*03:01 encompasses the information of HLA-DQA1*05:05 and serves as a strong genetic predictor of IFX treatment persistence and immunogenicity in Japanese patients with IBD, offering a potential biomarker for personalized therapy.

**Supplementary Information:**

The online version contains supplementary material available at 10.1007/s00535-026-02354-z.

## Introduction

Inflammatory bowel diseases (IBDs), including Crohn’s disease (CD) and ulcerative colitis (UC), are chronic, relapsing inflammatory disorders of the gastrointestinal tract with complex and heterogeneous etiologies [1, 2]. A variety of advanced therapies have recently been introduced for the treatment of IBD, such as anti-tumor necrosis factor (TNF) antibodies, anti-α4β7 integrin antibody, anti-interleukin-12/23 antibodies, Janus kinase inhibitors, and sphingosine-1-phosphate receptor modulators [3]. Selecting the most appropriate therapeutic agent for each individual patient remains a major clinical challenge. Delayed selection of the optimal treatment can result in prolonged impairment of quality of life, the development of complications, and, in some cases, the need for bowel resection. Among advanced therapies, anti-TNF agents—particularly infliximab (IFX)—were the first to be approved and continue to play a central role in IBD treatment [4, 5].

Nevertheless, primary non-response (PNR) and secondary loss of response (LOR) to IFX remain major obstacles to long-term disease control [6, 7]. These treatment failures are often attributed to the formation of anti-drug antibodies (ADAs), which can neutralize IFX or accelerate its clearance, thereby reducing therapeutic efficacy. Identifying patients at risk for PNR or early LOR before initiating IFX could improve treatment strategies and clinical outcomes.

Host genetic factors, including human leukocyte antigen (HLA) genotypes, are increasingly recognized as potential predictors of treatment response and immunogenicity to biologics. Among them, the HLA-DQA1*05 allele is associated with ADA development and reduced drug persistence in European populations [8]. Similarly, in Japanese patients with CD, HLA-DQA1*05 is associated with decreased persistence of IFX treatment [9]. However, there is substantial interethnic variation in genetic susceptibility to IBD, and the allele frequency of HLA-DQA1*05 in Europeans (approximately 40%) differs markedly from that in East Asians, including Japanese (approximately 8–9%) [4, 10]. Despite this, no studies have comprehensively evaluated the association between long-term IFX persistence and a broad range of HLA alleles—including HLA-DQA1*05—in East Asian IBD populations. Furthermore, in the context of HLA analysis for IFX treatment, HLA allele analysis at the 4-digit level, rather than at the 2-digit level, can be important. Ternette et al. demonstrated that among DQA1*05 alleles, both DQA1*05:01 and DQA1*05:05 are associated with the development of ADAs and reduced therapeutic efficacy of IFX [11]. Given the different allele frequencies of DQA1*05 in Japanese populations, the analyses of correlations among IFX persistence, ADA formation, and high-resolution 4-digit HLA alleles may lead to the identification of novel predictive markers of IFX treatment response.

In this study, we investigated associations among comprehensive HLA genotypes and IFX treatment persistence in a Japanese cohort of patients with IBD. We also analyzed the relationship between HLA alleles and ADA formation one year after IFX initiation. By integrating clinical, pharmacological, and genetic data, this study examined host-related factors influencing IFX efficacy and immunogenicity, with the aim of contributing to the development of personalized treatment strategies for Japanese patients with IBD.

## Methods

### Study design and ethics statements

This retrospective, observational cohort study was conducted at a single center in Japan (Tohoku University Hospital). The study protocol was reviewed and approved by the Ethics Committee of Tohoku University Hospital (2020-1-608). Written informed consent was obtained from all participants. The study was conducted in accordance with the Ethical Guidelines for Medical and Health Research Involving Human Subjects issued by the Japanese Ministry of Health, Labour and Welfare.

### Subjects

Ethnically Japanese patients with CD or UC who had previously received IFX (Remicade®, Mitsubishi Tanabe Pharma, Tokyo, Japan) at Tohoku University Hospital were consecutively enrolled between January 2002 and February 2025. Diagnoses of CD and UC were made based on the criteria established by the Japanese Ministry of Health, Labour and Welfare, and incorporated endoscopic, radiological, and histological findings.

A total of 301 patients with IBD for whom clinical and 4-digit HLA data were available were included in the study. The cohort comprised 219 patients with CD and 82 with UC. All patients were naïve to other biologics and JAK inhibitors—including adalimumab, golimumab, vedolizumab, ustekinumab, risankizumab, mirikizumab, tofacitinib, filgotinib, and upadacitinib—prior to the initiation of IFX. Clinical data were extracted from medical records. The duration from IFX initiation to discontinuation because of PNR or LOR was calculated. The discontinuation of IFX was defined as withdrawal of IFX therapy, including cases after dose escalation or interval shortening. PNR was defined as lack of clinical improvement at the end of induction therapy (week 14). LOR was defined as recurrence of disease activity after an initial clinical response during maintenance therapy. Patients were followed from the start of IFX treatment until treatment discontinuation because of PNR or LOR or the end of follow-up. Discontinuation because of PNR or LOR was considered an event, defined as withdrawal of IFX based on insufficient efficacy, determined from biochemical, clinical, or endoscopic findings, or because of the need for surgery due to disease progression. Discontinuations for reasons other than PNR or LOR (e.g., adverse events) were treated as censored observations. The reasons for IFX discontinuation were determined by the treating physician.

### Protocol of IFX administration

IFX was administered to patients with moderate to severe CD or UC whose inflammation was not adequately controlled with 5-aminosalicylic acid preparations, corticosteroids, or thiopurines. During the induction phase, IFX was given at a dose of 5 mg/kg at weeks 0, 2, and 6. This was followed by maintenance therapy with 5 mg/kg administered every 8 weeks for UC. For CD, in cases with insufficient response to maintenance therapy with 5 mg/kg every 8 weeks, dose escalation (10 mg/kg) or interval shortening (5 mg/kg every 4 weeks) was applied.

### Clinical factors investigated in this analysis

The following clinical variables were assessed: gender, disease type (CD or UC), age at diagnosis, disease duration at the start of IFX therapy, body mass index (BMI) at the start of IFX therapy, disease location (ileal, colonic, or ileocolonic), disease behavior (inflammatory, stricturing, or penetrating), presence of perianal disease (including perianal fistulas, abscesses, anal ulcers, and stenosis), history of intestinal resection, extent of UC (proctitis, left-sided colitis, or pancolitis), concomitant thiopurine use, serum albumin levels at the start of IFX therapy, C-reactive protein (CRP) levels at the start of IFX therapy, and HLA-DQA1*05 carrier status.

Age at IBD onset, disease duration at the initiation of IFX therapy, BMI, CRP, and serum albumin levels were treated as continuous variables. For survival analysis, these variables were categorized as follows. Age at IBD onset was dichotomized at 17 years according to the Montreal classification. BMI was categorized based on clinically accepted cutoffs: < 18 kg/m^2^ (underweight), 18–25 kg/m^2^ (normal), and > 25 kg/m^2^ (overweight). CRP and serum albumin were dichotomized using cutoffs of 0.3 mg/dL and 3.5 g/dL, respectively. Disease duration was dichotomized using the median value of 7 years as the cutoff.

### Genotyping and HLA imputation

Details of the methods used for DNA extraction and genotyping have been described previously [12]. The genomic DNA was isolated from peripheral blood leukocytes and genotyped using the Japonica Array V1 (Toshiba, Tokyo, Japan) [13], which is specifically designed for genotyping and imputation of the Japanese population. Single nucleotide polymorphism (SNP) quality control was performed prior to imputation, including the removal of variants with call rates of < 98%, a minor allele frequency < 1%, and a Hardy–Weinberg equilibrium of *p* < 1E-06.

SNP data from 301 samples were extracted from the extended major histocompatibility complex region (chr6: 28,510,120–33,480,577, hg38). Three-field HLA imputation of eight HLA genes (-A, -C, -B, -DRB1, -DQA1, -DQB1, -DPA1, -DPB1) was performed using the HIBAG R package with a previously constructed Japanese reference panel consisting of 418 Japanese healthy individuals [14]. Post-imputation quality control was applied by filtering samples with a call threshold of < 0.5 to remove low-confidence imputations.

### Anti-drug–antibody analysis

One year after the initiation of IFX treatment, venous blood samples were collected. Serum was isolated by centrifugation at 2000 g for 10 min and stored at − 80 °C until analysis. Free antibodies against IFX were measured using the IDKmonitor® Infliximab total ADA enzyme-linked immunosorbent assay from Immundiagnostik AG (Bensheim, Germany), with a detection cutoff for ADAs of 10 AU/mL. The serum samples were available for 83 patients treated with IFX.

### Statistical analysis

**Fig. 1:**
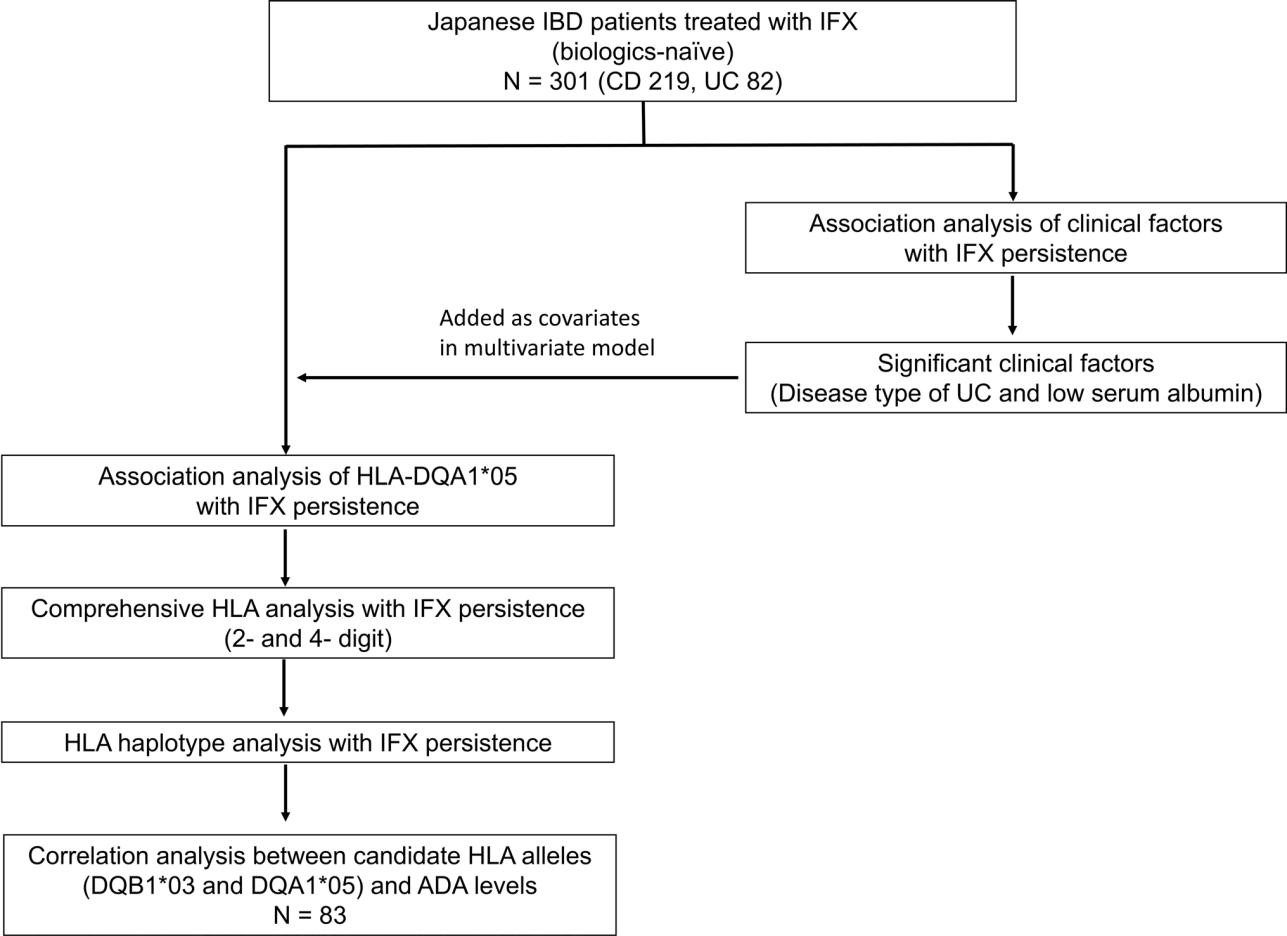
Flowchart of the study design. IBD, inflammatory bowel disease; IFX, infliximab; HLA, human leukocyte antigen; ADA, anti-drug-antibody

The study design is summarized in Fig. 1. For univariate analysis of the treatment persistence of IFX based on clinical factors, the log-rank test was used. Multivariate analysis was performed using a Cox proportional hazards model. A *p* value of < 0.05 was considered statistically significant. To assess genetic factors associated with IFX persistence, we examined the relationship between HLA-DQA1*05 and treatment persistence of IFX. These rates were evaluated using both the log-rank test and a Cox proportional hazards model. Disease type and baseline serum albumin levels were included as covariates in the Cox model because they were significantly associated with IFX persistence (*p* < 0.05).

HLA alleles with a frequency > 0.05 were included in the HLA-wide analyses. The treatment persistence for each HLA allele was calculated using Cox proportional hazards models adjusted for disease type and baseline serum albumin levels. A dominant genetic model, which is commonly used in HLA association studies, was applied. Under this model, individuals carrying at least one copy of the allele of interest were compared with those carrying none. For HLA-wide significance testing, nominal *p*-values were multiplied by the number of alleles tested at each HLA locus (Bonferroni correction). An adjusted *p*-value of < 0.05 was considered statistically significant.

To evaluate whether concomitant thiopurine therapy modified the association between candidate HLA alleles and IFX persistence, an interaction term between candidate HLA alleles and thiopurine use was included in the Cox proportional hazards model. Disease type and baseline serum albumin levels were included as covariates. Hazard ratios for thiopurine co-therapy stratified by HLA carrier status were estimated using estimated marginal means derived from the fitted model.

For the ADA analysis, the Wilcoxon rank-sum test was used to evaluate the association between ADA titers and the presence of each HLA allele. To assess the relationship between HLA alleles and ADA levels dichotomized using a cutoff of 10, Fisher’s exact test was applied.

Power calculations were conducted using the R package powerSurvEpi (version 0.1.3, https://CRAN.R-project.org/package=powerSurvEpi) to evaluate the adequacy of the sample size for detecting an association between HLA alleles and IFX persistence. Assuming an allele frequency of 5%, a hazard ratio (HR) of 2.0 and a total sample size of 301 patients, the statistical power was approximately 95.5%, indicating that the study was adequately powered to detect a clinically meaningful difference. All statistical analyses were performed using R software (version 4.5.1, https://www.r-project.org/).

## Results

### Patients’ characteristics and baseline data

Table 1 summarizes the baseline characteristics of the Japanese IBD patients. The median age at initial diagnosis was 22 years and the median disease duration at the start of IFX therapy was 6.3 years. Sixty-nine patients (23.5%) had a BMI of less than 18.0. The BMI of 188 patients (63.9%) ranged from 18 to 25, while 37 patients (12.6%) had a BMI greater than 25 (BMI data were unavailable for seven patients).

**Table 1:** Baseline characteristics and factors associated with the risk of infliximab discontinuation ^a^

Characteristic	*N*	Univariate		Multivariate	
		*P*-value	HR (95% CI)	*P*-value	HR (95% CI)
Total	301				
Sex					
Male (%)	201 (66.8)	0.44	1.14 (0.81–1.61)		
Female (%)	100 (33.2)	–	–		
Disease type					
Crohn's disease (%)	219 (72.8)	–	–		
Ulcerative colitis (%)	82 (27.2)	1.32E-03	1.84 (1.27–2.68)	1.09E-05	2.47 (1.65–3.69)
Age at diagnosis (years; Median (interquartile range))	22 (18–30)				
< 17 years old (%)	58 (19.3)	0.62	0.91 (0.63–1.32)		
Disease duration at the start of IFX therapy (years; Median (interquartile range))	6.3 (2.1–12.3)				
> 7 years (%)	140 (46.5)	0.31	0.85 (0.62–1.16)		
BMI (Median (interquartile range))	20.2 (18.1–22.8)				
> 18.0 (%)	69 (23.5)	0.31	1.20 (0.85–1.70)		
18.0–25.0 (%)	188 (63.9)	0.81	0.96 (0.70–1.32)		
< 25.0 (%)	37 (12.6)	0.36	0.80 (0.49–1.30)		
Location of Crohn's disease					
L1 (%)	30 (13.7)	0.76	1.08 (0.66–1.77)		
L2 (%)	36 (16.4)	0.04	0.59 (0.35–0.98)		
L3 (%)	153 (69.9)	0.13	1.35 (0.92–1.99)		
Behavior of Crohn's disease					
B1 (%)	82 (37.4)	0.63	0.91 (0.63–1.32)		
B2 (%)	94 (42.9)	0.19	0.79 (0.55–1.12)		
B3 (%)	43 (19.6)	0.02	1.66 (1.10–2.50)		
Previous intestinal resection of Crohn's disease(Yes, %)	128 (58.4)	0.30	0.83 (0.58–1.18)		
Perianal disease of Crohn's disease(Yes, %)	121 (55.3)	0.25	1.23 (0.87–1.74)		
Extent of Ulcerative colitis					
E1 (%)	7 (8.5)	0.31	0.48 (0.11–2.00)		
E2 (%)	24 (29.3)	0.06	0.47 (0.21–1.03)		
E3 (%)	51 (62.2)	0.01	2.54 (1.21–5.36)		
Concomitant thiopurine (Yes, %)	70 (23.3)	0.57	0.90 (0.62–1.30)		
Concomitant thiopurine dose (mg/kg; Median (interquartile range))	0.84 (0.65–1.01)				
Serum albumin levels at the baseline (g/dl; Median (interquartile range))	3.7 (3.2–4.0)				
< 3.5 g/dL (%)	125 (41.5)	7.62E-05	1.85 (1.37–2.52)	2.95E-05	2.14 (1.50–3.06)
CRP levels at the baseline (mg/dl; Median (interquartile range))	0.6 (0.1–1.9)				
> 0.3 mg/dL (%)	197 (65.4)	0.02	1.48 (1.05–2.08)	0.47	1.15 (0.79–1.67)
HLA-DQA1*05 carrier (Yes,%)	65 (21.6)	2.06E-04	1.97 (1.38–2.83)		

^a^*HR* hazard ratio, *CI* confidence interval, *IFX* infliximab, *BMI* body mass index, *CRP* C-reactive protein, *HLA* human leukocyte antigen

The study cohort consisted of 219 patients (72.8%) with CD and 82 (27.2%) with UC. The distribution of disease location was as follows: 30 patients (13.7%) had ileal disease, 36 (16.4%) had colonic disease, and 153 (69.9%) had ileocolonic disease. Regarding disease behavior, 82 patients (37.4%) had inflammatory type, 94 (42.9%) had stricturing type, and 43 (19.6%) had penetrating (fistulizing) type. Perianal lesions were observed in 121 patients (55.3%), and 128 patients (58.4%) had a history of intestinal resection. Among patients with UC, the extent of disease was classified as proctitis in seven cases (8.5%), left-sided colitis in 24 cases (29.3%), and total colitis in 51 cases (62.2%). Concomitant thiopurine therapy was administered to 70 patients (23.3%), with a median dose of 0.84 mg/kg. Sixty-five patients (21.6%) were identified as HLA-DQA1*05 carriers. At baseline, the median serum albumin and CRP levels were 3.7 g/dL and 0.3 mg/dL, respectively.

### Clinical factors associated with IFX persistence

Among 301 IBD patients, 167 cases (130 CD and 37 UC) discontinued IFX treatment due to disease recurrence. In UC patients, all cases had been treated with the standard protocol (5 mg/kg every 8 weeks). In CD patients, IFX was discontinued in 77 cases (59.2%) after the standard protocol, in 51 cases (39.2%) after dose escalation, and in two cases (1.6%) after interval shortening. The details of IFX administration protocols and outcomes for CD patients are summarized in supplementary Table 1.

The results of univariate and multivariate analyses assessing the association between clinical factors and the cumulative treatment persistence of IFX therapy are presented in Table 1. In the univariate analysis, UC (HR = 1.84, *p* = 1.32E-03), fistulizing disease behavior (HR = 1.66, *p* = 0.02), total colitis (HR = 2.54, *p* = 0.01), baseline serum albumin < 3.5 g/dL (HR = 1.85, *p* = 7.62E-05), and baseline CRP > 0.3 mg/dL (HR = 1.48, *p* = 0.02) were identified as risk factors for early discontinuation of IFX. In contrast, colonic-type CD (HR = 0.59, *p* = 0.04) was associated with prolonged IFX persistence. In the multivariate analysis, UC (HR = 2.47, *p* = 1.09E-05) and baseline serum albumin < 3.5 g/dL (HR = 2.14, *p* = 2.95E-05) remained significant independent predictors of early IFX discontinuation. Conversely, gender, age at diagnosis, disease duration at the start of IFX, BMI, history of intestinal resection, presence of perianal disease, and concomitant thiopurine use were not significantly associated with IFX discontinuation.

### Association between HLA-DQA1*05 and IFX persistence

**Fig. 2:**
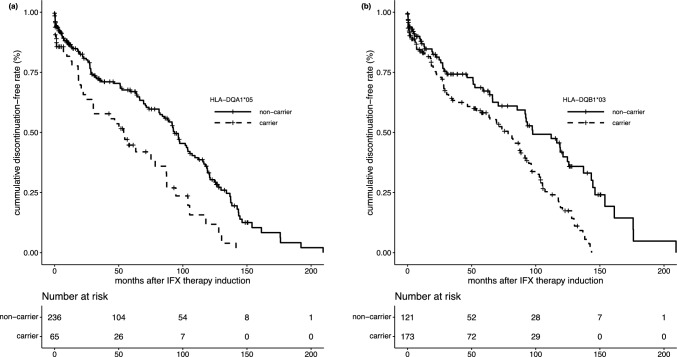
Kaplan–Meier curves for IFX discontinuation for the HLA-DQA1*05 (**a**) and HLA-DQB1*03 (**b**) genotypes. Numbers at risk are shown below the plots

The allele frequency of HLA-DQA1*05 in our cohort was 10.2%, which is comparable with that reported in the general Japanese population [9]. HLA-DQA1*05 was significantly associated with early discontinuation of IFX in the univariate analysis (HR = 1.97, *p* = 2.06E-04). The Kaplan–Meier curve for HLA-DQA1*05 is presented in Fig. 2a. Importantly, this association remained significant in the multivariate analysis (Table 2), which included baseline serum albumin < 3.5 g/dL and UC as covariates (HR = 2.03, *p* = 1.39E-04).

**Table 2:** Multivariate analysis of the association between HLA-DQA1*05 and the risk of infliximab discontinuation ^a^

Variables	N	*P*-value	HR (95% CI)
HLA-DQA1*05 carrier	65	1.39E-04	2.03 (1.41–2.92)
Serum albumin levels at the baseline < 3.5 g/dL	125	1.94E-06	2.22 (1.60–3.08)
Disease type			
Crohn's disease	219	–	–
Ulcerative colitis	82	2.59E-06	2.63 (1.76–3.94)

^a^*HR* hazard ratio, *CI* confidence interval, *HLA* human leukocyte antigen

We subsequently conducted separate analyses of the association for both CD and UC patients. These results are summarized in Supplementary Tables 2 and 3, respectively. Among patients with CD, univariate analysis showed that HLA-DQA1*05 carriers experienced significantly earlier discontinuation of IFX (HR = 2.25, *p* = 5.41E-05). This association remained significant in the multivariate analysis, which included baseline serum albumin < 3.5 g/dL, colonic-type CD, and fistulizing behavior as covariates (HR = 2.25, *p* = 1.32E-04). For patients with UC, univariate analysis showed that although the association did not reach statistical significance (HR = 1.81, *p* = 0.14), HLA-DQA1*05 carriers tended to have a worse prognosis compared with non-carriers. In the multivariate model, which included baseline serum albumin < 3.5 g/dL and total colitis as covariates, carriers of HLA-DQA1*05 tended to discontinue IFX earlier (HR = 2.23, *p* = 0.07). Kaplan–Meier curves for the analysis of CD and UC patients are shown in Supplementary Fig. 1a and b, respectively.

### Comprehensive analysis of HLA association with IFX persistence

For comprehensive HLA analysis, we included 40 alleles at 2-digit resolution and 52 alleles at 4-digit resolution, each with an allele frequency (AF) of 5% or higher. The details of the number of alleles analyzed for each HLA locus are provided in Supplementary Table 4. Each HLA allele was evaluated using multivariate analysis, adjusting for baseline serum albumin levels and disease type. The results of the 2-digit and 4-digit resolution analyses are summarized in Tables 3 and 4, respectively.

**Table 3:** HLA-wide analysis between each HLA type and the risk of infliximab discontinuation (2-digit resolution, allele frequency > 0.05 and *p*-value < 0.05)^a^

HLA type	Allele frequency	Carrier rate	*p*-value	HR (95% CI)
HLA-DQB1*03	0.35	0.59	1.89E-06	2.39 (1.67–3.43)
HLA-DQA1*05	0.11	0.22	1.39E-04	2.03 (1.41–2.92)
HLA-DQB1*04	0.21	0.38	0.01	0.64 (0.46–0.89)
HLA-B*46	0.08	0.15	0.02	1.71 (1.09–2.70)
HLA-A*02	0.28	0.48	0.02	1.46 (1.05–2.02)
HLA-A*11	0.09	0.17	0.03	0.60 (0.38–0.95)
HLA-DRB1*09	0.13	0.25	0.04	1.49 (1.02–2.16)
HLA-B*44	0.06	0.12	0.04	0.56 (0.32–0.99)

^a^*HLA* human leukocyte antigen, *HR* hazard ratio, *CI* confidence interval

**Table 4:** HLA-wide analysis between each HLA type and the risk of infliximab discontinuation (4-digit resolution, allele frequency > 0.05 and *p*-value < 0.05)^a^

HLA type	Allele frequency	Carrier rate	*p*-value	HR (95% CI)
HLA-DQB1*03:01	0.14	0.26	9.42E-05	2.03 (1.42–2.90)
HLA-DQA1*05:05	0.07	0.13	4.42E-04	2.18 (1.41–3.36)
HLA-A*02:07	0.06	0.12	0.01	2.03 (1.22–3.39)
HLA-DQB1*04:02	0.08	0.15	0.01	0.56 (0.36–0.88)
HLA-DQA1*03:02	0.14	0.25	0.02	1.58 (1.09–2.28)
HLA-B*46:01	0.08	0.15	0.02	1.71 (1.09–2.70)
HLA-DQA1*03:03	0.19	0.35	0.03	0.68 (0.48–0.96)
HLA-A*11:01	0.09	0.17	0.03	0.60 (0.38–0.95)
HLA-DQB1*03:03	0.14	0.25	0.03	1.48 (1.03–2.13)
HLA-DRB1*09:01	0.13	0.25	0.04	1.49 (1.02–2.16)
HLA-B*51:01	0.12	0.21	0.04	1.47 (1.01–2.15)

^a^*HLA* human leukocyte antigen, *HR* hazard ratio, *CI* confidence interval

In the 2-digit resolution analysis, only HLA-DQB1*03 and HLA-DQA1*05 remained statistically significant after correction for multiple testing. Notably, HLA-DQB1*03 demonstrated the strongest association with IFX persistence (AF = 0.35, carrier rate = 0.59, HR = 2.39, *p* = 1.89E-06), exceeding that of HLA-DQA1*05 (HR = 1.99, *p* = 3.91E-04). The Kaplan–Meier curve for HLA-DQB1*03 is presented in Fig. 2b. In addition, we performed stratified analyses of HLA-DQB1*03 separately in patients with CD and UC, with the results summarized in Supplementary Tables 5 and 6. HLA-DQB1*03 showed a significant association with IFX persistence in both diseases; however, similar to HLA-DQA1*05, the association was stronger in CD than in UC (HR = 2.21, p = 2.17E-04 for CD and HR = 2.25, p = 0.02 for UC). Kaplan–Meier curves for CD and UC are shown in Supplementary Fig. 2a and b, respectively.

**Fig. 3:**
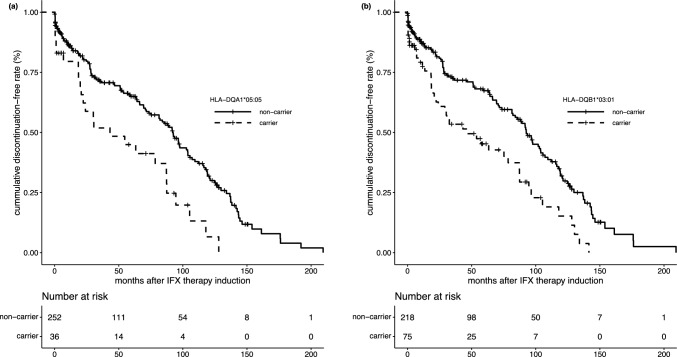
Kaplan–Meier curves for IFX discontinuation for the HLA-DQA1*05:05 (a) and HLA-DQB1*03:01 (b) genotypes. Numbers at risk are shown below the plots

In the 4-digit resolution analysis, HLA-DQB1*03:01 exhibited the strongest association (AF = 0.14, carrier rate = 0.26, HR = 2.03, *p* = 9.42E-05), followed by HLA-DQA1*05:05 (AF = 0.07, carrier rate = 0.13, HR = 2.18, *p* = 4.42E-04). In the Japanese population, HLA-DQA1*05:05 constitutes the majority of HLA-DQA1*05 alleles, while other alleles are rare. In our population, the allele frequencies of HLA-DQA1*05 subtypes were: DQA1*05:01—0.00%, DQA1*05:03—1.56%, DQA1*05:05—6.75%, DQA1*05:07—1.73%, and DQA1*05:08—0.52%. These findings are generally consistent with previously reported HLA allele frequencies in the Japanese population [15]. The Kaplan–Meier curves for HLA-DQA1*05:05 and HLA-DQB1*03:01 are presented in Fig. 3a and b, respectively. Among the HLA-DQB1*03 alleles, HLA-DQB1*03:03 was also associated with IFX persistence (AF = 0.14, carrier rate = 0.25, HR = 1.48, *p* = 0.03), while HLA-DQB1*03:02 showed no significant association (AF = 0.07, carrier rate = 0.14, HR = 1.28, *p* = 0.25). However, after correction for multiple testing, only HLA-DQB1*03:01 and HLA-DQA1*05:05 showed significant association.

### The effect of thiopurine co-therapy on the genetic risks for IFX persistence

We evaluated the impact of thiopurine co-therapy on IFX persistence across the relevant genetic risk groups (HLA-DQA1*05, HLA-DQB1*03, HLA-DQA1*05:05, and HLA-DQB1*03:01). The results are summarized in Supplementary Fig. 3 and 4 and Supplementary Table 7. Although thiopurine use tended to improve IFX persistence irrespective of the HLA allele status, none of the associations reached statistical significance. Moreover, formal interaction testing revealed no significant effect modification by thiopurine co-therapy for any of the examined HLA alleles. These findings indicate that, in our cohort, thiopurine co-therapy did not mitigate the genetic risks associated with these HLA alleles.

### Association between HLA haplotypes and IFX persistence

The comprehensive HLA association analysis identified HLA-DQB1*03 and HLA-DQA1*05 as having particularly strong associations with IFX persistence. Therefore, we performed haplotype analyses focusing on combinations of *HLA-DQB1* and *HLA-DQA1* alleles in relation to IFX persistence (Supplementary Tables 8 and 9).

In 2-digit resolution haplotype analysis, the combination of HLA-DQB1*03 and HLA-DQA1*05 showed the strongest association with IFX persistence (HR = 2.06, *p* = 1.85E-04). At 4-digit resolution, the haplotype consisting of HLA-DQB1*03:01 and HLA-DQA1*05:05 exhibited the strongest association (HR = 2.18, *p* = 3.38E-04). In all HLA-DQA1*05:05 allele carriers, the corresponding haplotype was HLA-DQB1*03:01. In contrast, approximately half of the HLA-DQB1*03:01 carriers formed haplotypes with non-DQA1*05:05 alleles, and these non-DQA1*05:05 haplotypes were also associated with early discontinuation of IFX therapy (HR = 1.82, *p* = 0.02, Supplementary Table 10).

Consistent with the findings of the HLA-wide analysis, the haplotype comprising HLA-DQA1*05:01 and HLA-DQB1*02:01—which strongly influences IFX persistence in European cohorts [16]—was not detected in our Japanese cohort.

### Correlation of ADA levels with HLA-DQA1*05 and HLA-DQB1*03

Finally, we performed a correlation analysis between the two HLA alleles that were significantly associated with IFX persistence—HLA-DQA1*05 and HLA-DQB1*03—and ADA levels measured one year after IFX initiation. The results are summarized in Supplementary Table 11.

Eighty-three serum samples were available for this analysis. HLA-DQA1*05 was significantly associated with both higher ADA levels (*p* = 1.26E-03) and increased ADA positivity (*p* = 0.02), whereas HLA-DQB1*03 showed no such association (*p* = 0.43 and 0.82, respectively). At 4-digit resolution, both HLA-DQB1*03:01 and HLA-DQA1*05:05—alleles previously associated with IFX persistence—also showed significant associations with ADA responses. HLA-DQB1*03:01 was associated with higher ADA levels (*p* = 3.23E-03) and showed a trend toward increased ADA positivity (*p* = 0.06). HLA-DQA1*05:05 was significantly associated with both ADA levels (*p* = 3.54E-03) and ADA positivity (*p* = 0.02). Notably, 4-digit DQB1*03 alleles other than HLA-DQB1*03:01, including HLA-DQB1*03:02 and DQB1*03:03, did not show a significant association with ADA levels (Supplementary Table 11). These findings are consistent with the lack of a significant association between HLA-DQB1*03 and ADA levels observed in the two-digit analysis.

In addition, we compared ADA levels between the HLA-DQA1*05:05–HLA-DQB1*03:03 haplotype and the non–HLA-DQA1*05:05–HLA-DQB1*03:03 haplotype but no significant difference was observed (*p* = 0.44).

## Discussion

We have examined associations between HLA types across the wide HLA region and IFX persistence at both 2-digit and 4-digit resolutions in an East Asian population. We restricted our analysis to patients who had never received advanced therapy, thereby minimizing possible confounding effects of prior biologic treatments and enabling a more accurate evaluation of factors influencing IFX persistence. Low serum albumin levels, UC diagnosis, CD behavior, and total colitis in UC were identified as clinical parameters associated with early IFX discontinuation. As a genetic factor, HLA-DQA1*05 was strongly associated with IFX persistence, consistent with previous reports. Moreover, we identified that HLA-DQB1*03, particularly HLA-DQB1*03:01, was strongly associated with IFX persistence and that ADA titers were strongly associated with these HLA types.

Serum albumin plays a crucial role in the transport and metabolism of many drugs by binding to them with high affinity, thereby slowing their elimination rates [17, 18]. High serum albumin levels have been reported to be associated with lower clearance, longer half-life, and higher IFX concentrations [19], while, in our study, low baseline serum albumin levels were associated with early IFX discontinuation. Consistently, several previous studies have also shown that low baseline serum albumin predicts non-response to anti-TNF therapy [20, 21].

Among clinical factors, UC diagnosis was associated with reduced IFX persistence, consistent with previous findings of higher discontinuation rates in patients with acute severe UC [22]. Furthermore, several studies have shown that IFX is discontinued earlier in UC than in CD [23–27]. In addition, TNF plays a central role in CD pathogenesis, whereas multiple inflammatory pathways, including but not limited to TNF, are involved in UC [28, 29]. These differences may influence the effectiveness and persistence of IFX therapy. Furthermore, during the treatment of CD, IFX dose escalation or interval shortening can be implemented when clinically indicated. This treatment flexibility may be one of the reasons for the higher IFX persistence observed in patients with CD.

Total colitis in UC and fistulizing disease behavior in CD were also associated with early discontinuation of IFX. The extent of UC influences the clinical course and prognosis of the disease [30, 31]. Patients with extensive inflammation are more likely to require intensive therapies or even colectomy. Similarly, fistulizing disease behavior in CD is associated with poor response to IFX and the need for alternative approaches, such as surgical or endoscopic intervention [32, 33], which is consistent with our findings.

As a genetic factor involved in IFX persistence, HLA-DQA1*05 showed a significant association. The association between HLA-DQA1*05 and IFX persistence and the AF difference of HLA-DQA1*05 between Japanese and European populations is consistent with multiple previous reports [4, 9, 10, 34, 35]. Four-digit resolution analysis in European populations showed HLA-DQA1*05:01 and HLA-DQA1*05:05 to be significantly associated with IFX persistence [8]. However, in Japan, the AF of HLA-DQA1*05:01 is extremely low (0.07%) [15], whereas it is approximately 20–30% in Europe and the United States [36–38]. Given the low AF of HLA-DQA1*05:01 in Japan, other HLA alleles may serve as better predictors of IFX persistence.

In addition to confirming the association with DQA1*05, we identified a novel strong association between DQB1*03 and IFX persistence in Japanese IBD patients. These genetic effects were more evident in CD than in UC, which may reflect both disease-specific treatment strategies and differences in sample size between the two disease subtypes. Moreover, at 4-digit resolution analysis, HLA-DQB1*03:01 was the most strongly associated allele with IFX persistence. HLA-DQB1*03 encodes the β chain of the HLA-DQ molecule, a class II HLA molecule involved in recognizing exogenous and self-antigens and presenting them to CD4⁺ T cells to initiate immune responses [39]. Although HLA-DQB1*03 and DQB1*03:01 have been implicated in several autoimmune diseases and drug-related adverse events [40–42], no previous studies have reported a strong association with IBD.

In our analysis, concomitant thiopurine therapy did not improve IFX persistence, regardless of genetic risk status. This finding contrasts with previous reports from Western populations [8]. A plausible explanation for this discrepancy is the relatively low dose of thiopurines used in our cohort (median, 0.84 mg/kg). In East Asian populations, including Japanese individuals, the NUDT15 R139C variant is highly prevalent and has been reported to increase susceptibility to thiopurine-induced leukopenia [43]. As a result, thiopurine doses commonly used in Japan (approximately 1–2 mg/kg) are lower than those typically administered in Western populations (2.0–2.5 mg/kg) [44, 45]. In our analysis, although not statistically significant, thiopurine co-therapy showed a trend toward a protective effect on IFX persistence. These findings suggest that the use of adequately dosed thiopurines may still have the potential to mitigate genetic risk and improve IFX persistence in Japanese patients, warranting further investigation.

The haplotype analysis showed that HLA-DQB1*03:01–HLA-DQA1*05:05 exhibited the strongest association. A previous European study demonstrated that the haplotypes HLA-DQA1*05:01–HLA-DQB1*02:01 and HLA-DQA1*05:05–HLA-DQB1*03:01 were associated with IFX persistence. In the Japanese population, however, the AF of HLA-DQB1*02:01 is only 0.13%, indicating that the HLA-DQA1*05:05–HLA-DQB1*03:01 haplotype may be more relevant [16]. While all carriers of HLA-DQA1*05:05 also possessed HLA-DQB1*03:01, individuals with HLA-DQB1*03:01 in the absence of HLA-DQA1*05:05 still showed associations with early LOR, indicating that HLA-DQB1*03:01 is a stronger clinical marker of IFX treatment persistence than HLA-DQA1*05:05 in the Japanese population.

The candidate HLA alleles, HLA-DQA1*05:05 and HLA-DQB1*03:01, are both significantly associated with ADA levels 1 year after the initiation of IFX treatment. Although the analysis was limited by a small sample size and requires further investigation, the findings indicate that HLA-DQB1*03:01 may reduce IFX persistence by promoting the production of ADA. Considering these findings, HLA-DQB1*03:01, which encompasses the associations of HLA-DQA1*05:05, appears to be a promising predictive marker for IFX persistence in Japanese populations.

This study has several limitations that should be considered. First, it was a retrospective, single-center study. Second, the number of patients with UC was smaller than that of patients with CD, limiting our ability to adequately evaluate the utility of the candidate HLA markers specifically in UC. Finally, serum samples were not available for all patients in the cohort, resulting in a limited and fragmented analysis of ADA levels. In particular, ADA measurements were not performed in patients who discontinued IFX within the first year, precluding evaluation of the genetic contribution to early ADA formation. These limitations can be addressed by large-scale, multi-center validation studies.

In conclusion, our study provides novel evidence that HLA-DQB1*03:01 is a strong genetic predictor of IFX persistence in Japanese patients with IBD, potentially outperforming the widely recognized marker, HLA-DQA1*05:05. These findings highlight the importance of population-specific genetic backgrounds for the optimization of biologic therapies and underscore the need for incorporating HLA genotyping into personalized treatment strategies for IBD.

## Supplementary Information

Below is the link to the electronic supplementary material.

Supplementary file1 (DOCX 716 KB)
