# Supplementary material for: HLA-DQB1*03:01 and HLA-DQA1*05:05 as key genetic determinants of infliximab response and immunogenicity in Japanese patients with inflammatory bowel disease
Source: J Gastroenterol. 2026 Feb 6;61(5):547–58. doi: 10.1007/s00535-026-02354-z (PMC13157412; doi:10.1007/s00535-026-02354-z)
Supplement: Supplementary file 1 — Supplementary file1 (DOCX 716 KB) [file 535_2026_2354_MOESM1_ESM.docx]

**article title:** HLA-DQB1*03:01 and HLA-DQA1*05:05 as key genetic determinants of infliximab response and immunogenicity in Japanese patients with inflammatory bowel disease

**journal name:** Journal of Gastroenterology

**author names:** Ryuya Osaka^1^, Takeo Naito*^1^, Seik-Soon Khor^2,3^, Yoichi Kakuta^1^, Yosuke Kawai^2^, Masao Nagasaki^4,5^, Hiroshi Meguro^1^, Hideya Iwaki^1^, Daisuke Okamoto^1^, Hiroshi Nagai^1^, Yusuke Shimoyama^1^, Rintaro Moroi^1^, Hisashi Shiga^1^, Yoshitaka Kinouchi^6^, Atsushi Masamune^1^

**affiliation:**

^1^Division of Gastroenterology, Tohoku University Graduate School of Medicine, Sendai, Japan.

^2^Genome Medical Science Project, National Center for Global Health and Medicine, Tokyo, Japan

^3^Singapore Centre for Environmental Life Sciences Engineering, Nanyang Technological University, Singapore, Singapore.

^4^Division of Biomedical Information Analysis, Medical Research Center for High Depth Omics, Medical Institute of Bioregulation, Kyushu University, Japan

^5^Center for Genomic Medicine, Graduate School of Medicine, Kyoto University, Kyoto, Japan

^6^Student Health Care Center, Institute for Excellence in Higher Education, Tohoku University, Sendai, Japan

**e-mail address of the corresponding author:** [takeo.naito.b3@tohoku.ac.jp](mailto:takeo.naito.b3@tohoku.ac.jp)

Supplementary Table 1. Summary of IFX administration protocols and outcomes in Crohn’s disease patients

| Patterns of IFX administration protocols and outcomes | Number of cases (total CD patients = 219) |
| --- | --- |
| Observation ended without IFX discontinuation | 89 |
| Observation ended after the standard protocol | 88 (98.9%) |
| Observation ended after dose escalation | 1 (1.1 %) |
| IFX discontinued due to disease recurrence | 130 |
| IFX discontinued after the standard protocol | 77 (59.2 %) |
| IFX discontinued after dose escalation | 51 (39.2 %) |
| IFX discontinued after interval shortening | 1 (0.8 %) |
| IFX discontinued after dose escalation and subsequent interval shortening | 1 (0.8 %) |

IFX, infliximab

Standard protocol: IFX maintenance therapy with 5 mg/kg administered every 8 weeks

Dose escalation: IFX maintenance therapy with 10 mg/kg administered every 8 weeks

Interval shortening: IFX maintenance therapy with 5 mg/kg administered every 4 weeks

Supplementary Table 2. Association between HLA-DQA1*05 and the risk of infliximab discontinuation in Crohn's disease in multi-variate analysis

| Variables | N | P-value | HR (95% CI) |
| --- | --- | --- | --- |
| HLA-DQA1*05 carrier | 49 | 1.32E-04 | 2.25 (1.49-3.42) |
| Serum albumin levels at the baseline  < 3.5 g/dL | 94 | 1.72E-03 | 1.76 (1.24-2.52) |
| Location |  |  |  |
| L2 | 36 | 0.07 | 0.61 (0.36-1.03) |
| Behavior |  |  |  |
| B3 | 43 | 0.01 | 1.77 (1.17-2.70) |

HLA, human leukocyte antigen; HR, hazard ratio; CI, confidence interval

Supplementary Table 3. Association between HLA-DQA1*05 and the risk of infliximab discontinuation in Ulcerative colitis in multi-variate analysis

| Variables | N | P-value | HR (95% CI) |
| --- | --- | --- | --- |
| HLA-DQA1*05 carrier | 16 | 0.07 | 2.23 (0.95-5.23) |
| Serum albumin levels at the baseline  < 3.5 g/dL | 31 | 5.72E-04 | 3.54 (1.73-7.28) |
| Extent |  |  |  |
| E3 | 51 | 0.01 | 2.90 (1.30-6.47) |

HLA, human leukocyte antigen; HR, hazard ratio; CI, confidence interval

Supplementary Table 4. The numbers of alleles with allele frequency of 5% or higher

| HLA type | the number of alleles (2-digit resolution) | the number of alleles (4-digit resolution) |
| --- | --- | --- |
| HLA-A | 6 | 8 |
| HLA-C | 6 | 7 |
| HLA-B | 7 | 8 |
| HLA-DRB1 | 7 | 6 |
| HLA-DQA1 | 3 | 7 |
| HLA-DQB1 | 4 | 8 |
| HLA-DPA1 | 2 | 3 |
| HLA-DPB1 | 5 | 5 |

HLA, human leukocyte antigen

Supplementary Table 5. Association between HLA-DQB1*03 and the risk of infliximab discontinuation in Crohn's disease in multi-variate analysis

| Variables | N | P-value | HR (95% CI) |
| --- | --- | --- | --- |
| HLA-DQB1*03 carrier | 133 | 2.17E-04 | 2.21 (1.45-3.37) |
| Serum albumin levels at the baseline  < 3.5 g/dL | 94 | 4.06E-03 | 1.94 (1.34-2.80) |
| Location |  |  |  |
| L2 | 36 | 0.42 | 0.80 (0.45-1.39) |
| Behavior |  |  |  |
| B3 | 43 | 0.01 | 1.73 (1.13-2.66) |

HLA, human leukocyte antigen; HR, hazard ratio; CI, confidence interval

Supplementary Table 6. Association between HLA-DQB1*03 and the risk of infliximab discontinuation in Ulcerative colitis in multi-variate analysis

| Variables | N | P-value | HR (95% CI) |
| --- | --- | --- | --- |
| HLA-DQB1*03 carrier | 40 | 0.02 | 2.25 (1.14-4.46) |
| Serum albumin levels at the baseline  < 3.5 g/dL | 31 | 1.22E-04 | 4.00 (1.97-8.11) |
| Extent |  |  |  |
| E3 | 51 | 3.47E-03 | 3.21 (1.47-7.01) |

HLA, human leukocyte antigen; IFX, infliximab; HR, hazard ratio; CI, confidence interval

Supplementary Table 7. Effect of thiopurine co-therapy on the risk of infliximab discontinuation across risk HLA allele groups

| HLA type | Carrier status | P-value  for thiopurine co-therapy | HR (95% CI)  for thiopurine co-therapy | P-value  for interaction |
| --- | --- | --- | --- | --- |
| DQA1*05 | non-carrier | 0.058 | 0.63 (0.39 - 1.02) |  |
|  | carrier | 0.27 | 0.67 (0.33 - 1.36) | 0.87 |
| DQB1*03 | non-carrier | 0.42 | 0.77 (0.41 - 1.45) |  |
|  | carrier | 0.44 | 0.81 (0.49 - 1.36) | 0.88 |
| DQA1*05:05 | non-carrier | 0.061 | 0.64 (0.40 - 1.02) |  |
|  | carrier | 0.15 | 0.55 (0.24 - 1.24) | 0.72 |
| DQB1*03:01 | non-carrier | 0.13 | 0.67 (0.40 - 1.12) |  |
|  | carrier | 0.23 | 0.67 (0.36 - 1.29) | 0.99 |

HLA, human leukocyte antigen; IFX, infliximab; HR, hazard ratio; CI, confidence interval

Supplementary Table 8. Haplotype analysis between each combination of HLA-DQA1 and HLA-DQB1 locus, and the risk of infliximab discontinuation (2-digit resolution, allele frequency > 0.05 and P-value < 0.05)

| haplotype | frequency | P-value | HR (95% CI) |
| --- | --- | --- | --- |
| HLA-DQA1*05 ~ HLA-DQB1*03 | 0.11 | 1.85E-04 | 2.06 (1.41-3.01) |
| HLA-DQA1*03 ~ HLA-DQB1*03 | 0.22 | 2.56E-03 | 1.68 (1.20-2.35) |
| HLA-DQA1*03 ~ HLA-DQB1*04 | 0.17 | 0.01 | 0.63 (0.44-0.91) |
| HLA-DQA1*01 ~ HLA-DQB1*06 | 0.34 | 0.36 | 0.85 (0.61-1.20) |
| HLA-DQA1*01 ~ HLA-DQB1*05 | 0.10 | 0.91 | 1.02 (0.68-1.53) |

HLA, human leukocyte antigen; IFX, infliximab; HR, hazard ratio; CI, confidence interval

Supplementary table 9. Haplotype analysis between each combination of HLA-DQA1 and HLA-DQB1 locus, and the risk of infliximab discontinuation (4-digit resolution, allele frequency > 0.05 and P-value < 0.05)

| haplotype | frequency | P-value | HR (95% CI) |
| --- | --- | --- | --- |
| HLA-DQA1*05:05 ~ HLA-DQB1*03:01 | 0.08 | 3.38E-04 | 2.18 (1.42-3.34) |
| HLA-DQA1*03:02 ~ HLA-DQB1*03:03 | 0.14 | 0.02 | 1.58 (1.09-2.29) |
| HLA-DQA1*03:03 ~ HLA-DQB1*04:01 | 0.14 | 0.13 | 0.74 (0.51-1.09) |
| HLA-DQA1*01:02 ~ HLA-DQB1*06:02 | 0.07 | 0.16 | 0.69 (0.42-1.16) |
| HLA-DQA1*03:01 ~ HLA-DQB1*03:02 | 0.07 | 0.18 | 1.34 (0.87-2.07) |
| HLA-DQA1*01:03 ~ HLA-DQB1*06:01 | 0.21 | 0.33 | 0.84 (0.59-1.19) |
| HLA-DQA1*01:04 ~ HLA-DQB1*05:03 | 0.06 | 0.96 | 1.01 (0.62-1.65) |

HLA, human leukocyte antigen; IFX, infliximab; HR, hazard ratio; CI, confidence interval

Supplementary table 10. Haplotype analysis of HLA-DQA1 alleles paired with HLA-DQB1*03:01 and the risk of infliximab discontinuation.

| haplotype | frequency | proportion within DQB1*03:01 haplotypes (%) | P-value | HR (95% CI) |
| --- | --- | --- | --- | --- |
| HLA-DQA1*05:05 ~ HLA-DQB1*03:01 | 0.08 | 57.1 | 3.38E-04 | 2.18 (1.42-3.34) |
| Non- HLA-DQA1*05:05 ~ HLA-DQB1*03:01 | 0.06 | 42.9 | 0.02 | 1.82 (1.10-3.03) |
| HLA-DQA1*03:03 ~ HLA-DQB1*03:01 | 0.01 | 8.6 |  |  |
| HLA-DQA1*05:03 ~ HLA-DQB1*03:01 | 0.02 | 12.8 |  |  |
| HLA-DQA1*05:07 ~ HLA-DQB1*03:01 | 0.02 | 12.8 |  |  |
| HLA-DQA1*05:08 ~ HLA-DQB1*03:01 | 0.01 | 4.3 |  |  |
| HLA-DQA1*06:01 ~ HLA-DQB1*03:01 | 0.01 | 4.3 |  |  |

HLA, human leukocyte antigen; IFX, infliximab; HR, hazard ratio; CI, confidence interval

Supplementary table 11. Association between each HLA type and anti-drug-antibody level

| HLA type | Carrier status | ADA level Median (interquartile range) | P-value Wilcoxon rank sum test | Hodges–Lehmann estimate (95% CI) | P-value Fisher's exact test |
| --- | --- | --- | --- | --- | --- |
| 2-digits |  |  |  |  |  |
| HLA-DQA1*05 | non-carrier | 8.6 (8.0 - 12.9) |  |  |  |
|  | carrier | 57.9 (9.9 - 120.0) | 1.26E-03 | 48.1 (1.73 - 101.2) | 0.02 |
| HLA-DQB1*03 | non-carrier | 9.2 (8.3–15.7) |  |  |  |
|  | carrier | 8.4 (7.9–18.6) | 0.43 | -0.27 (-0.94 - 0.57) | 0.82 |
| 4-digits |  |  |  |  |  |
| HLA-DQA1*05:05 | non-carrier | 8.7 (8.0 - 13.7) |  |  |  |
|  | carrier | 70.6 (53.5 - 94.9) | 3.54E-03 | 50.1 (31.9 - 110.1) | 0.02 |
| HLA-DQB1*03:01 | non-carrier | 8.7 (8.0 - 13.3) |  |  |  |
|  | carrier | 49.1 (9.6 - 115.2) | 3.23E-03 | 32.5 (1.1 - 78.0) | 0.06 |
| HLA-DQB1*03:02 | non-carrier | 9.1 (8.2 - 16.2) |  |  |  |
|  | carrier | 8.1 (7.9 - 11.7) | 2.00E-01 | -0.51 (-2.40 - 0.22) | 0.75 |
| HLA-DQB1*03:03 | non-carrier | 8.7 (7.9 - 18.7) |  |  |  |
|  | carrier | 8.0 (7.7 - 9.2) | 1.40E-01 | -0.60 (-3.18 - 0.20) | 0.35 |

HLA, human leukocyte antigen; ADA, anti-drug-antibody

Supplementary Fig. 1. Kaplan–Meier curve of IFX persistence stratified by HLA-DQA1*05 carrier status in (a) Crohn’s disease, (b) Ulcerative colitis.


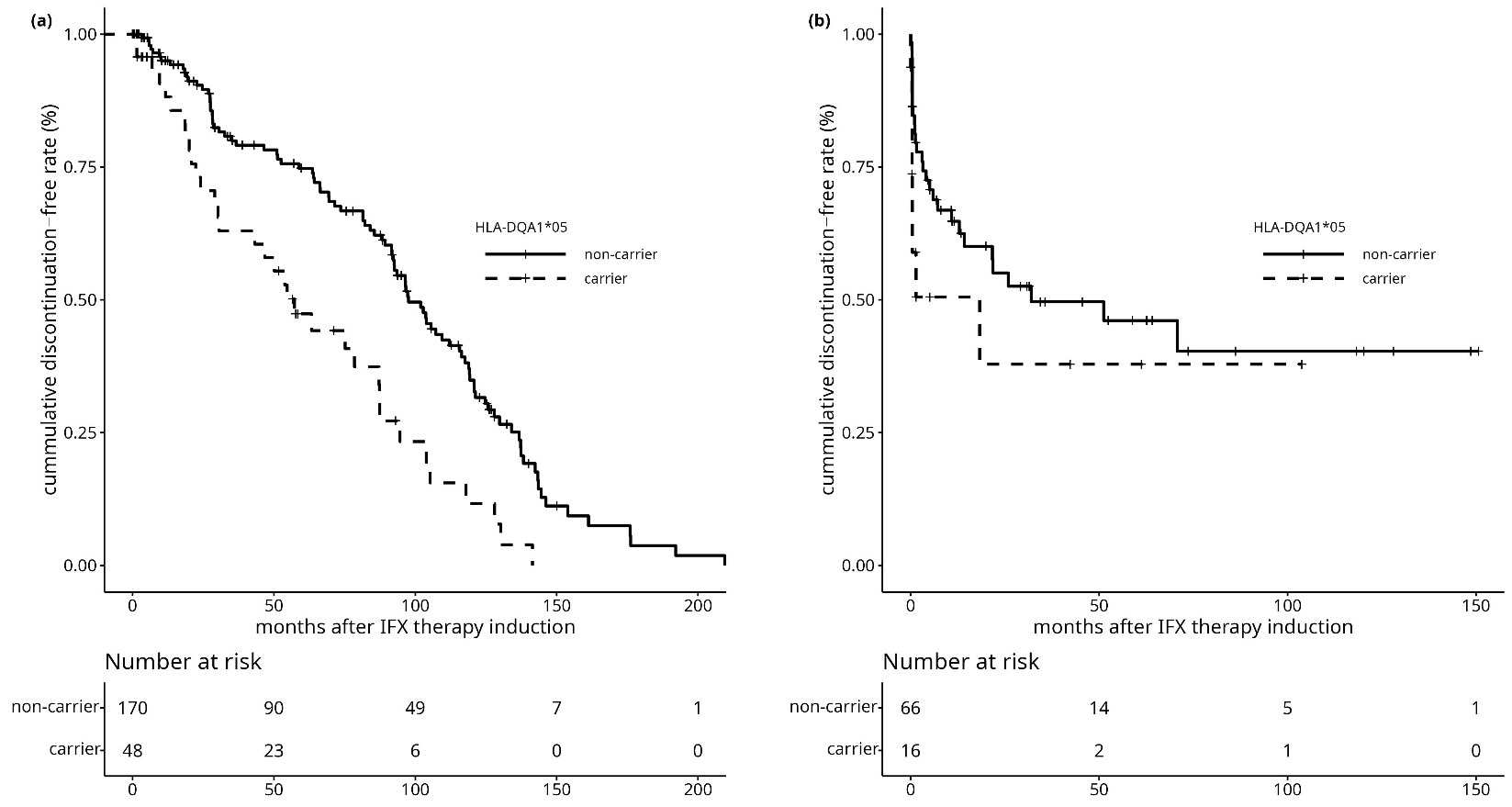


IFX, infliximab; HLA, human leukocyte antigen

Supplementary Fig. 2. Kaplan–Meier curve of IFX persistence stratified by HLA-DQB1*03 carrier status in (a) Crohn’s disease, (b) Ulcerative colitis.


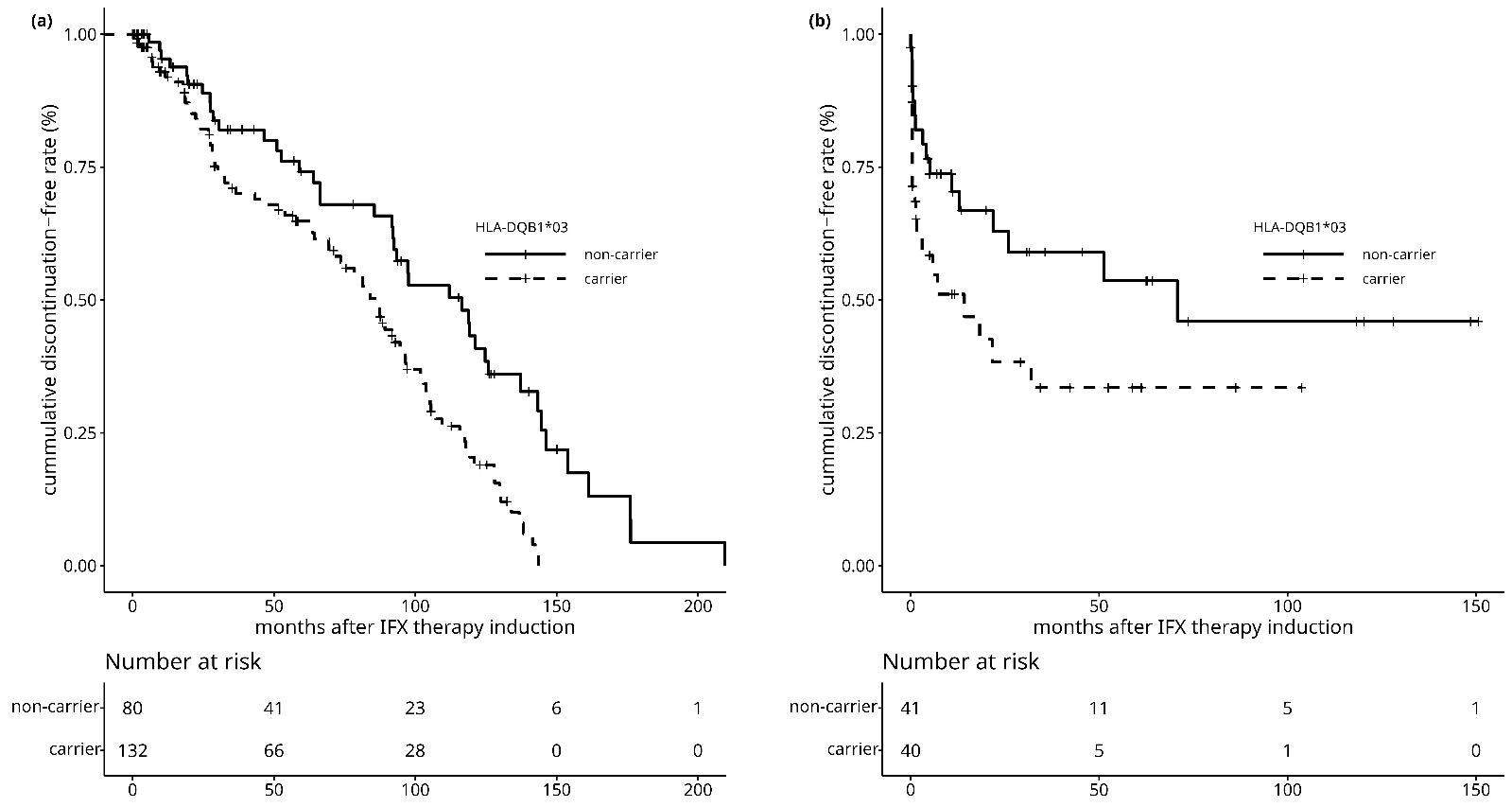


IFX, infliximab; HLA, human leukocyte antigen

Supplementary Fig. 3. Kaplan–Meier curve of IFX persistence stratified by HLA-DQA1*05/HLA-DQB1*03 carrier status and thiopurine co-therapy.


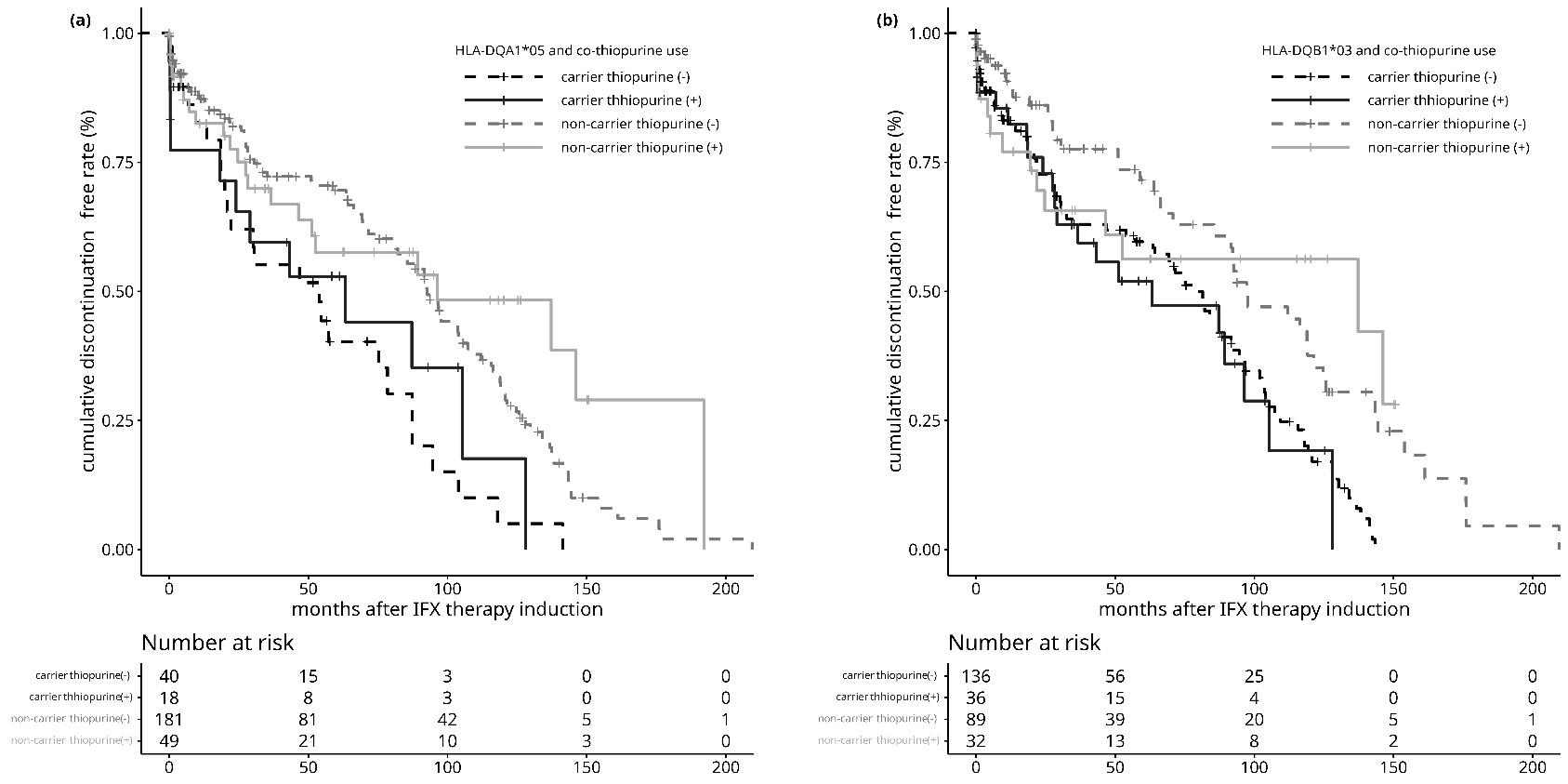


IFX, infliximab; HLA, human leukocyte antigen

Supplementary Fig. 4. Kaplan–Meier curve of IFX persistence stratified by HLA-DQA1*05:05/HLA-DQB1*03:01 carrier status and thiopurine co-therapy.


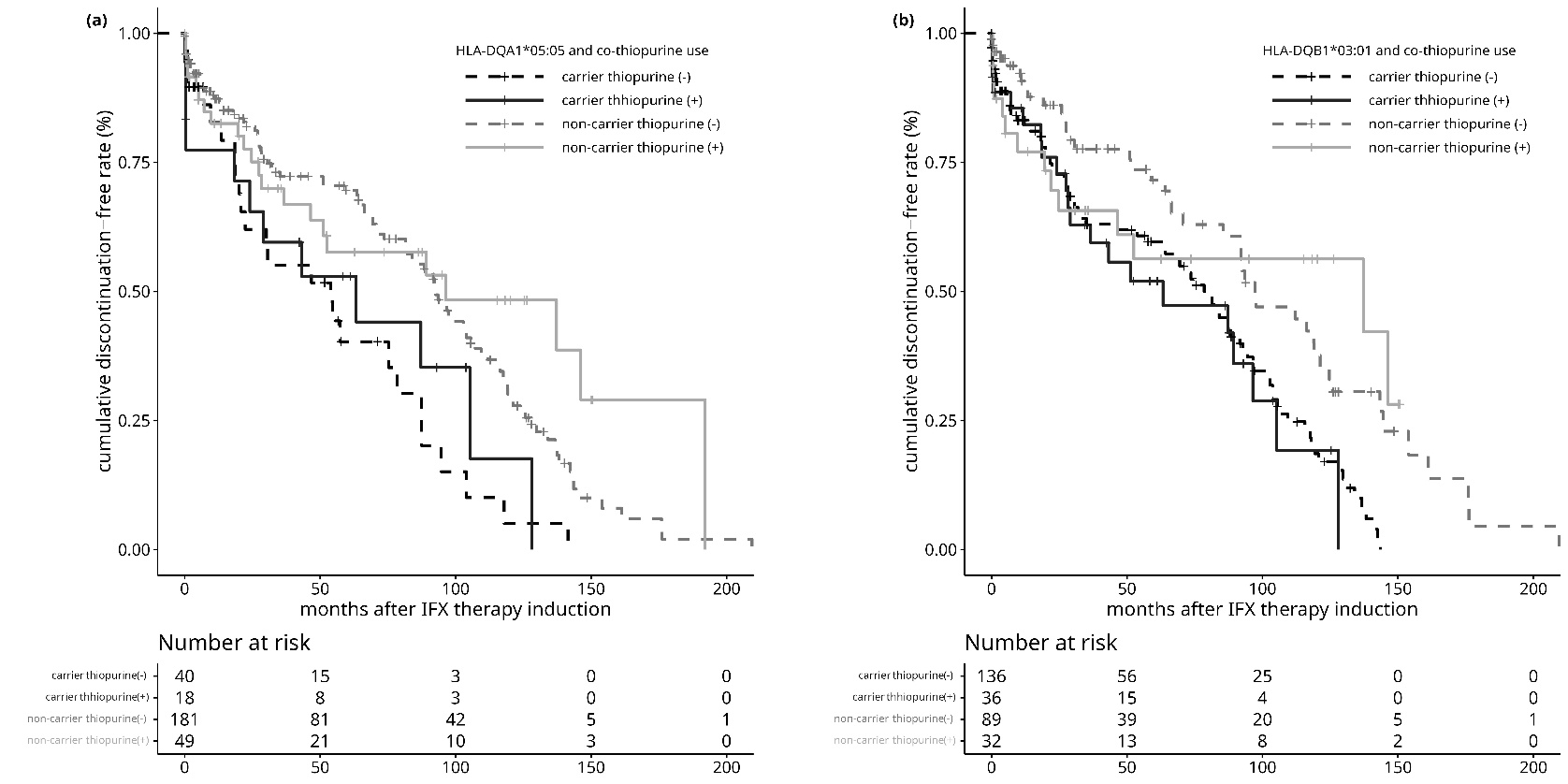


IFX, infliximab; HLA, human leukocyte antigen
